# Effects of Enactment in Episodic Memory: A Pilot Virtual Reality Study with Young and Elderly Adults

**DOI:** 10.3389/fnagi.2014.00338

**Published:** 2014-12-17

**Authors:** Najate Jebara, Eric Orriols, Mohamed Zaoui, Alain Berthoz, Pascale Piolino

**Affiliations:** ^1^UMR 7152 CNRS, Collège de France, Paris, France; ^2^Institut de Psychologie, Université Paris Descartes, Paris, France; ^3^INSERM U894, Centre de Psychiatrie et Neurosciences, Paris, France; ^4^Institut Universitaire de France, Paris, France

**Keywords:** episodic memory, binding, aging, virtual reality, everyday memory, action, decision, events memory

## Abstract

None of the previous studies on aging have tested the influence of action with respect to the degree of interaction with the environment (active or passive navigation) and the source of itinerary choice (self or externally imposed), on episodic memory (EM) encoding. The aim of this pilot study was to explore the influence of these factors on feature binding (the association between what, where, and when) in EM and on the subjective sense of remembering. Navigation in a virtual city was performed by 64 young and 64 older adults in one of four modes of exploration: (1) *passive condition* where participants were immersed as passengers of a virtual car [no interaction, no itinerary control (IC)], (2) *IC* (the subject chose the itinerary, but did not drive the car), (3) *low*, or (4) *high navigation control* (the subject just moved the car on rails or drove the car with a steering-wheel and a gas pedal on a fixed itinerary, respectively). The task was to memorize as many events encountered in the virtual environment as possible along with their factual (what), spatial (where), and temporal (when) details, and then to perform immediate and delayed memory tests. An age-related decline was evidenced for immediate and delayed feature binding. Compared to passive and high navigation conditions, and regardless of age-groups, feature binding was enhanced by low navigation and IC conditions. The subjective sense of remembering was boosted by the IC in older adults. Memory performance following high navigation was specifically linked to variability in executive functions. The present findings suggest that the decision of the itinerary is beneficial to boost EM in aging, although it does not eliminate age-related deficits. Active navigation can also enhance EM when it is not too demanding for subjects’ cognitive resources.

## Introduction

Episodic memory (EM) contains specific events of one’s life and enables humans to travel back in personal time to re-experience events. Retrieval depends on the successful “recollection” of the features of the original event, such as the time, place, people, emotional, and idiosyncratic as well as sensorimotor aspects of that event (Tulving, 2002). Much of what people remember in everyday life refers to actions they carried out in a complex environment. For instance, during the recall of a walk in the streets of a city, several components are associated in EM: *what* happened (e.g., “meeting Charles”), and the corresponding items referring to *elements* (buildings, people) of the environment; perceptual features (*details*); internal feelings, thoughts; spatial (“*where*”); and temporal (“*when*”) situation and also self-performed actions. The process of integrating the core content (*what happened*) with other contextual features of an event into a cohesive memory representation is a key feature of EM, and is designated as “*binding*.” This mechanism makes it possible to form connections that give a memory its specificity and distinctiveness (Johnson et al., 1993; Naveh-Benjamin, 2000; Hommel, 2004; Van Asselen et al., 2006; Kessels et al., 2007; Mitchell and Johnson, 2009).

Older adults often have difficulty retrieving specific events from their personal past, providing general information instead (Levine et al., 2002; Piolino et al., 2002, 2006, 2010; Martinelli et al., 2013). Moreover, in laboratory settings, they show difficulty in learning new items (Albert, 1994; Luo and Craik, 2008), and in determining in which experimental context they had encoded a previously encountered item [see Johnson et al. (1993) for review]. The memory for spatial context (Kessels et al., 2007) as well as for temporal context (Fabiani and Friedman, 1997) declines with age. Elderly people generally perform better on tests of item memory than on tests that require feature binding (Spencer and Raz, 1995; Chalfonte and Johnson, 1996; Mitchell et al., 2000; Kessels et al., 2007; Mitchell and Johnson, 2009). These deficits have been mainly associated with age-related effects on both the *associative* and *strategic* components of EM (Moscovitch, 1992; Shing et al., 2008; Piolino et al., 2010). The associative component refers to binding mechanisms based on the medial temporal lobe including the hippocampus (Zimmer et al., 2006). The strategic component refers to cognitive control processes based on prefrontal regions that monitor memory functions at both encoding and retrieval (Simons and Spiers, 2003).

One way to improve EM in laboratory settings is to give older participants encoding instructions that favor the link between a content and its context (Naveh-Benjamin et al., 2004, 2005; Glisky and Kong, 2008) or to add an environmental support at encoding that can serve as a compensatory strategy for deficient memory processing [see Glisky (2001), Naveh-Benjamin et al. (2002), and Luo and Craik (2008) for reviews]. One encoding strategy that has been considered as one of the most effective in recent decades is the enactment effect that consists in enhancing memory by linking implicitly or explicitly the information to be remembered with personal actions (Engelkamp et al., 1994; Engelkamp, 1998; Zimmer and Cohen, 2001; Earles and Kersten, 2002; Earles et al., 2004; Madan and Singhal, 2012).Typically, in action memory paradigms, memory for actions is enhanced if they are actually performed during encoding [subject-performed task (SPT)], compared to verbal encoding, even in older adults (Feyereisen, 2009). The mechanisms responsible for the enactment effect are still under debate. Some authors consider that the multimodal nature of a “movement” may reinforce item-specific information by enriching its encoding specificity, thereby acting as an additional retrieval cue (Engelkamp, 1998), while others argue that the benefit of action is due to the involvement of self goal-directed activities rather than motor activities *per se* (Kormi-Nouri, 1995). As action is always embedded in an intention to interact with the environment, rather than just a movement (Berthoz, 2003), acting in accordance with a decision therefore seems to benefit memory performances by improving the distinctiveness of mnesic traces (Viard et al., 2011; Voss et al., 2011).

Virtual reality is now considered as particularly relevant to test cognition in naturalistic and controlled situations with different levels of immersion (Bohil et al., 2011; Mueller et al., 2012; Zawadzki et al., 2013). Strong concordance with real-world abilities is a notable benefit of virtual reality (VR) technology (Schultheis et al., 2002; Plancher et al., 2010, 2012). Because the critical purpose of VR is to allow users to carry out cognitive and sensorimotor activities while being immersed in an artificial world (Schultheis et al., 2002; Fuchs et al., 2006), VR appears as a good tool to investigate the enactment effect on EM in complex situations (Brooks et al., 1999; Sauzéon et al., 2011; Plancher et al., 2012). Yet, VR-based research has long been used to study large-scale spatial skills in young (e.g., Maguire et al., 1997; Carassa et al., 2002; Lambrey et al., 2008; Galati et al., 2010; Iglói et al., 2010; Barra et al., 2012; Gras et al., 2013) and older adults (Lövden et al., 2005; Iaria et al., 2009; Moffat, 2009; Head and Isom, 2010; Bohbot et al., 2012; Klencklen et al., 2012; Gyselinck et al., 2013; Taillade et al., 2013), rather than the memory of complex episodes. While there has been a substantial amount of VR research on spatial learning comparing active vs. passive navigation, contradictory results have been reported, with some studies showing a positive effect of active navigation (Brooks et al., 1999; Péruch and Wilson, 2004; Wallet et al., 2011; Plancher et al., 2012), others reporting no benefits (Wilson, 1999; Gaunet et al., 2001; Foreman et al., 2005; Plancher et al., 2008), or even a negative effect (Sandamas and Foreman, 2003; Taillade et al., 2013).

In the domain of VR-based EM study, several researchers have used virtual immersion to assess item memory (e.g., objects) (Parsons and Rizzo, 2008; Sauzéon et al., 2011; Widman et al., 2012), or item memory in association with contextual information (e.g., the character, the location, and the moment associated with each object) (Burgess et al., 2001, 2002; Rauchs et al., 2008; Plancher et al., 2010, 2012, 2013). For instance, using a simulation of the California Verbal Learning Test in a virtual apartment (HOMES test), Arvind-Pala et al. (2014) confirmed poor recall, but better recognition, and intact clustering and proactive interference effects for item memory in older adults. In another study investigating age-related EM deficits of real-life events encountered in a virtual town, Plancher et al. (2010) showed that older adults recalled poorer episodic bindings compared to younger adults, regardless of the mode of encoding (intentional or incidental). Interestingly, some VR-based studies investigated the benefit of active navigation (compared to passive navigation) on subsequent EM performances. For instance, comparing participants assigned to a free active navigation using a joystick with participants in a passive condition, Brooks et al. (1999) found that the active condition improved the recall of spatial layout, but not the recall of objects seen during navigation. The authors attributed the benefit of action to an additional motor trace that increases specificity of the memory, but argued that it was limited to aspects of the virtual environment (VE) that are directly targeted by the interaction (here the spatial layout). More recently, Sauzéon et al. (2011) found that EM for objects placed in the rooms of two virtual apartments (HOMES test) was enhanced by active compared to passive navigation. Active participants had better item-specific measures such as learning and recognition, compared to passive participants, and they made fewer false recognitions. A similar beneficial effect of active navigation was reported by Plancher et al. (2012) in aging. Active navigation as a driver of a car, compared to passive navigation as a passenger, in the same participants, boosted feature binding (what–where–when) in healthy elderly participants and in patients with mild cognitive impairment and to a lesser extent in patients with Alzheimer’s disease. Nevertheless, there was a negative effect of active navigation on recall of perceptual details associated to elements (e.g., a car accident). Overall, the benefit of active navigation was assumed to result from the enrichment of item-specific processing (Sauzéon et al., 2011; Plancher et al., 2012, 2013), and the appropriateness of perceptive-motor traces at encoding for a specific memory task (Brooks et al., 1999; Wallet et al., 2011), while the detriment could depend on the level of complexity of the active navigation (Gaunet et al., 2001; Wilson and Peruch, 2002; Wolbers and Hegarty, 2010; Plancher et al., 2012). In some cases, active navigation might require additional cognitive resources that are not fully available for the encoding process, leading to a detrimental effect on some aspects of EM.

According to some authors (Wilson, 1999; Bakdash et al., 2008; Chrastil and Warren, 2012), the inconsistent results concerning the active–passive effect during virtual navigation on subsequent memory might be due to discrepancies in the experimental designs, notably with regard to the manipulation of control differing in terms of sensorimotor stimulation and its confounding effects with psychological activity (planning, decision-making, and attention). In this line of research, Bakdash et al. (2008) demonstrated in young adults that spatial performance was comparable when the VE was learned with decision-making in the absence of motor control or decision-making and motor control, but was much worse when only motor control was present. They concluded that decision-making is an essential cognitive process in active navigation that impacts spatial memory. A more recent VR-based EM study aspired to disentangle these two components of action on items and spatial memory in young adults (Plancher et al., 2013). It was found that both conditions enhanced subsequent spatial memory compared to passive navigation, but motor interaction produced worse memory for items, unlike decision-making.

In continuity with these two previous studies, the objective of the present one was to test how different components of action control at encoding (active navigation and decision) may influence feature binding EM performance (item plus context) and effect of aging. We used a computer-based simulation to manipulate the degree of interaction at encoding, and investigate subsequent EM in groups of young and older adults in: (1) a passive condition (where the subject was just immersed as the passenger of a car, i.e., no active navigation, no decision); (2) an itinerary condition (the subject was immersed as a passenger and chose the itinerary but did not drive the car); (3) a low active navigation condition (the subject moved the car on rails, but the itinerary was fixed); and (4) a high active navigation condition [the subject drove the car using the usual driving mode (a steering-wheel and pedals), but the itinerary was fixed]. The latter two navigation conditions differed in the degree of interactive sensorimotor engagement, but more especially they differed in the degree of executive/attentional load. On the one hand, higher navigation control adds sensorimotor interaction, which could help EM, but it also makes driving more complex in the VE, requiring a higher level of attentiveness (Blankertz et al., 2010) and thus could be detrimental for EM, especially in older adults. It has been shown that age-related memory differences after active navigation are mediated by executive functions (Taillade et al., 2013). On the other hand, the low navigation condition involves lower sensorimotor interaction, but it also adds an environmental support at encoding (driver assistance) that could compensate for deficient memory processing (Craik, 1986; Luo and Craik, 2008). Finally, given that the itinerary control (IC) condition involved only right/left decisions, it was assumed that this condition would engage the same amount of cognitive resources as the low active navigation condition. We thus mainly expected: (1) a large decline with aging for feature binding EM performance; (2) a beneficial effect for both age-groups of IC and low active navigation conditions, in comparison with the passive and the high active navigation conditions; and (3) a possible reduction of age-related decline in the decision and low active navigation conditions.

## Materials and Methods

### Participants

One hundred twenty-eight volunteers, 64 young, and 64 elderly adults (32 males and 32 females in each group, mean age 27 years, ranging from 19 to 40 years old for the young adults and a mean age of 65 years, ranging from 52 to 78 years old for the older adults) took part in the study. All had normal or corrected-to-normal vision and had a driving license. They provided written informed consent, and were paid for their participation. The local ethical committee of the CNRS approved the experimental protocol. Volunteers were divided into 4 groups of 16 in each age-group. All participants were tested individually and in only one condition.

All participants were unmedicated, living at home, and screened for absence of history of alcohol or substance abuse, head trauma, major disease affecting brain function, depression (BFS self-rating mood scale, Von Zerssen et al., 1974), and abnormal general cognitive functioning as assessed by the Mini Mental Scale (Folstein et al., 1975). Lastly, both age-groups were matched according to their verbal abilities and crystallized intelligence as assessed by the Mill Hill test (Deltour, 1993; a multiple-choice synonym vocabulary test).

### Brief cognitive assessment

To assess that general cognitive abilities were well matched across participants assigned to the four conditions, they were screened using a brief battery assessing cognitive functions that comprised: (1) The verbal and visual memory subscales of the clinical memory scale MEM-III (Wechsler, 2000). In the verbal memory subscale, participants heard a story and had to memorize its content. They then underwent immediate and delayed recalls. In the visual memory subscale, participants were asked to memorize pictures of visual scenes with different people doing different things. They then had to recall the elements making up the scene together with their spatial location, immediately and after a delay. We recorded two global scores, one of verbal memory (out of 50) and the other of visual memory (out of 64); (2) Working memory was assessed by computerized forward visuo-spatial span and short-term feature binding span (Picard et al., 2012). In the latter task, participants had to memorize increasingly long strings of objects associated with a specific spatial context (area of a grid) after having mentally associated the picture of an object displayed below the grid with its location in the grid according to a color code. We recorded a mean score of the two span tasks. (3) The Trail-Making Test (Lezak et al., 2004) was used as a measure of shifting. A score was computed by subtracting the response time for part A from part B. The results are presented in Table 1. Finally, the older participants were also screened for their subjective memory complaints (CDS, McNair and Kahn, 1983).

**Table 1:** **Description of the population as a function of age and experimental condition**.

	Experimental conditions								ANOVAs		
	Passive (1)		Itinerary control (2)		Low navigation control (3)		High navigation control (4)		Group effect	Condition effect	Interaction
	YA	OA	YA	OA	YA	OA	YA	OA	F(1,120)	F(3,120)	F(3,120)
Participants (*N*)	16 (8F; 8M)	16 (8F; 8M)	16 (8F; 8M)	16 (8F; 8M)	16 (8F; 8M)	16 (8F; 8M)	16 (8F; 8M)	16 (8F; 8M)			
Age	25.68 (2.91)	64.18 (6.67)	25.25 (4.66)	65.68 (6.94)	24.00 (2.55)	65.62 (5.77)	28.25 (6.76)	65.18 (8.02)	1452.95***	0.71	1.00
									η^2^ = 0.92	η^2^ = 0.01	η^2^ = 0.02
Mill Hill	36.12 (2.87)	37.75 (6.67)	32.91 (4.14)	35.87 (5.84)	36.31 (5.34)	37.00 (5.44)	33.93 (5.16)	34.81 (5.64)	5.27*	1.72	0.37
									η^2^ = 0.04	η^2^ = 0.04	η^2^ = 0.00
CDS	–	43.35 (21.01)	–	40.50 (14.55)	–	46.75 (17.33)	–	39.60 (18.87)		0.35	
										η^2^ = 0.01	
Verbal memory	29.93 (4.89)	23.43 (8.35)	28.50 (6.42)	24.31 (5.95)	32.18 (5.60)	23.37 (5.84)	27.81 (5.30)	24.81 (7.30)	21.18***	1.09	0.68
									η^2^ = 0.15	η^2^ = 0.02	η^2^ = 0.01
Visual memory	86.32 (14.50)	53.12 (22.86)	84.50 (20.30)	56.00 (23.14)	91.50 (15.91)	57.43 (13.51)	82.68 (22.25)	53.56 (17.81)	85.12***	0.64	0.17
									η^2^ = 0.41	η^2^ = 0.01	η^2^ = 0.00
Working memory	12.44 (1.99)	9.62 (2.18)	11.43 (1.63)	9.94 (2.20)	12.81 (1.47)	9.12 (1.41)	11.00 (1.59)	9.31 (1.78)	57.43***	1.76	2.56
									η^2^ = 0.32	η^2^ = 0.04	η^2^ = 0.06
Executive functions (TMT B-A, sec)	17.00 (10.65)	30.82 (16.72)	26.18 (10.41)	37.83 (27.47)	17.02 (6.18)	29.04 (16.86)	24.12 (13.99)	46.95 (37.93)	18.22***	1.16	0.50
									η^2^ = 0.13	η^2^ = 0.02	η^2^ = 0.01

***p < 0.001, **p < 0.01, *p < 0.05.

### Experimental VR episodic memory assessment (VR–EM test)

#### Material

**Figure 1:**
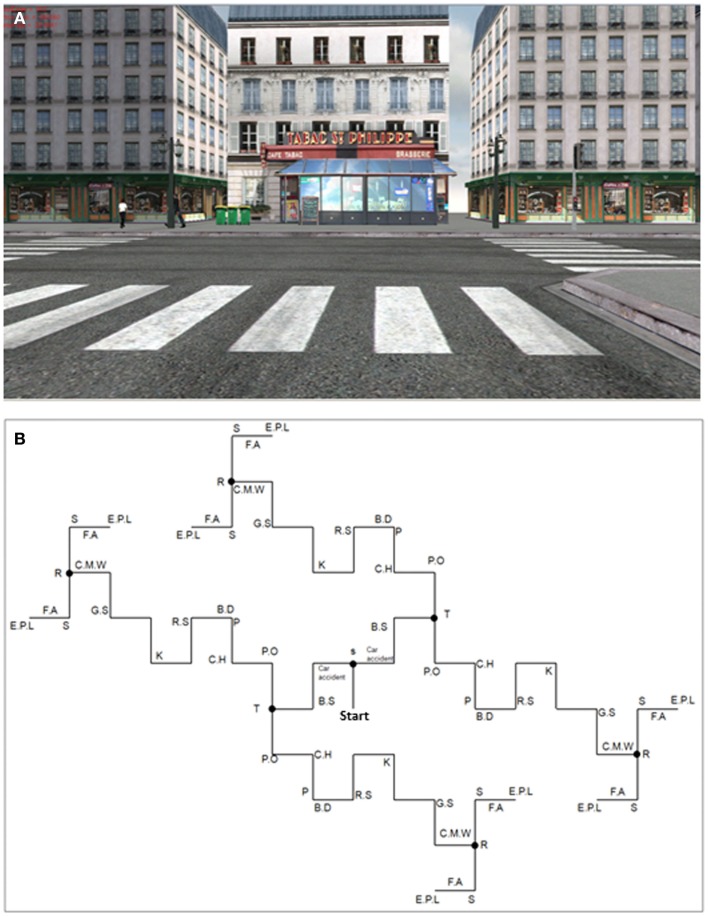
**Example of a screenshot (A) and map of the virtual town with main elements located on the map (B)**. Since the environment was built symmetrically, wherever “itinerary choice” participants turned they always saw the same elements. S, supermarket; car accident; B.S, bus stop; T, tobacco shop; P.O, post-office; C.H, city hall; P, public parking lot; B.D, business district; R.S, road safety sign; K, kebab shop; G.S, grocery store; C.M.W, two cars in the middle of the road; R, restaurants; S, train station; F.A, action against famine sign; E.P.L, external parking lot.

A virtual town (see an example of a view, Figure 1A) was built with Virtools Dev. 3.0 (http://www.virtools.com) and was projected via a PC (DELL PRECISION M6300) on a large SONY screen (Resolution 1932*1080) covering 66° of the visual field in a first-person perspective. The VE was projected 150 cm in front of the participants who were seated in a comfortable chair at the center of the screen.

To develop a full measure of EM retrieval, a rich virtual town was created by the Memory and Cognition laboratory at Paris-Descartes University (EditoMem and SimulMem). It was composed of buildings, people, cars, different typical objects of a town (barriers, lampposts, etc.), and several intersections (where participants could decide or were constrained to turn left or right) and background noise of the city. EM was solicited by an environment composed of 16 different scenes, such as the supermarket, the post office, the town hall, a car accident, representing the main landmarks and salient events, each scene comprising prominent associated elements (e.g., a man in a suit walking in front of the post office). The scenes were mainly located at the intersections when the participants were stopped at the traffic lights (for 5 s) or in the middle of the road (e.g., the car accident). The saliency of scenes and associated elements was validated based on our previous VR studies in young and older adults (Plancher et al., 2010, 2012, 2013). Importantly, the virtual town was built symmetrically (see Figure 1B) so that regardless of the direction taken by the subjects at the intersection (either a left or a right turn), they saw the same thing.

The material used for the driving was composed of a steering-wheel allowing control of the vehicle inside the VE, and the pedals allowing participants to control speed.

#### Procedure

Participants were tested individually. The experimenter was present and depending on the condition, sat either alongside or behind the participant (see the “Experimental conditions” section below).

Before the test session, the participants underwent a training session in an empty environment (with only streets), in order to familiarize them with the equipment and the VE with a different spatial layout from that of the town subsequently used for the test. The objectives of this training session were twofold: to provide participants with an initial experience of a VE, and to familiarize them with control of the virtual car. The training session lasted until participants felt familiar with the equipment; they were free to navigate anywhere in the training period.

**Figure 2:**
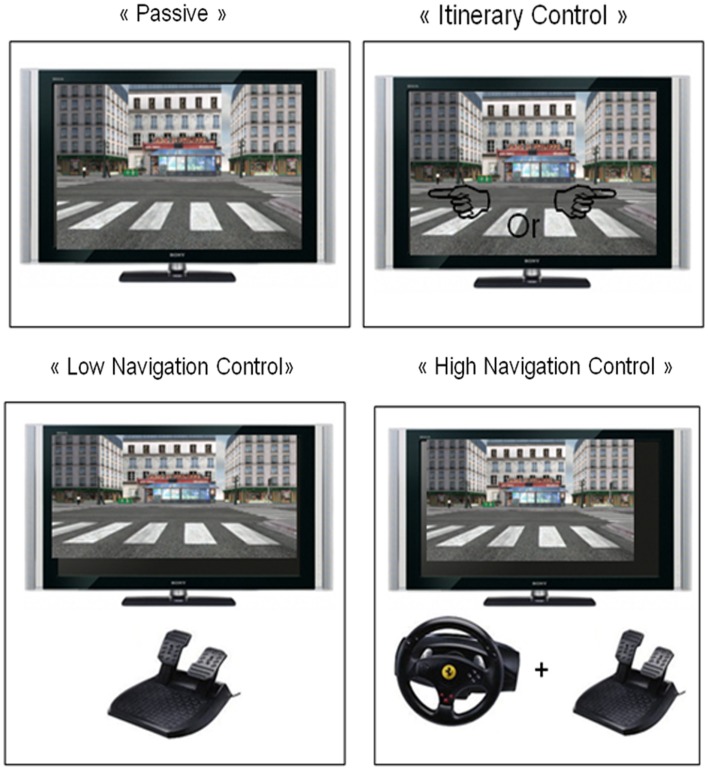
**The four experimental conditions assessed during the virtual exploration in the VR–EM test**.

Participants were then requested to explore the experimental environment by active or passive navigation (as the driver or passenger of a virtual car). Subjects were encouraged to pay attention to as much detail as possible (i.e., details, spatial locations, and temporal order) of the different scenes/events encountered during their navigation (“What,” “Where,” and “When”), since they would subsequently undergo a memory test (intentional encoding). An example of a scene not presented in the experiment was shown as a picture before the exploration, to ensure that the participants understood what they had to memorize. They were randomly assigned to one of the four conditions:

(1) A “passive” condition (memorize without driving or choosing the itinerary) where each participant was just a passenger while the experimenter drove the car. The visual information displayed to each passive subject was the same as that of a subject who interacted with the environment. This condition served as a baseline condition compared to the three “active” experimental conditions requiring different processes (itinerary or navigation control) (see Figure 2).
(2) An “IC” condition (memorize and choose the itinerary without driving) was tested. Participants were passengers of the car and could choose the direction (left or right), by verbal instructions to the experimenter, at each intersection. This condition required participants to interact with the VE just at a purely cognitive “decisional” level. In the following two conditions, participants were “physically active” as driver of the car, but they were requested to follow a given itinerary (indicated by the experimenter).
(3) A “Low Navigation Control (LNC)” condition (memorize and move the car on rails) in which each participant could displace the car on rails, manipulating only the pedals (a gas pedal to control the speed and a brake pedal to stop). Pressing the pedals controlled the speed but there was no enactment of the movement associated with the direction (such as turning the steering-wheel).
(4) A “High Navigation Control (HNC)”, condition (memorize and drive) similar to ordinary driving in which each participant drove as in real-life, manipulating a steering-wheel and pedals. He/she could interact with the environment by pressing pedals for speed control of the vehicle and by turning the steering-wheel to control the direction of the vehicle trajectory in the virtual town.

Finally, in order to better standardize subjects’ attention to different features of the environment in the different conditions (i.e., more time spent viewing this or that detail), each participant was informed that he/she would be stopped at each set of traffic lights until it changed to green (5 s). In the navigation conditions, participants were instructed to stop and restart their car at each traffic light. Driving speed was limited so that participants could neither drive above a set speed nor stop anywhere in the town apart from traffic lights.

#### VR episodic memory assessment (VR–EM test)

After exploring the virtual town, all participants underwent a series of memory tests previously validated in VR–EM studies comparing young and older adults after immersion in a VE (Plancher et al., 2010, 2012).

##### Free recall test

Immediately after the navigation, the participants were asked to verbally report the elements of the scenes/events encountered during their navigation (what) and indicate for each of them the different components (for 10 min):

– To test the memory of the content information (“what”), they were asked to try and remember each scene/event with associated elements and to give the most salient “*details*” accompanying these elements [e.g., “I saw a car crash between a *yellow* car and a *blue* car (event), a woman with a *blue t-shirt* witnessed the accident (salient element)”].
– For each scene/event previously recalled, the participants were requested to report its location from their viewpoint and if they had turned to the left or to the right after seeing it. They had to situate each scene roughly either at the beginning, the middle, or the end of the circuit, and then to report their temporal order to test the sequential order in which they met them. This allowed us to obtain information about the association between the visual perception of the scene and the spatial egocentric “where” and temporal “when” components.
– At the end of free recall, the Remember/Know paradigm (Tulving, 1985; Gardiner, 2001) was proposed asking participants if they remembered (or just knew) the details of their navigation. They were asked to provide the intensity of their sense of remembering (using an analogical scale from 0 to 5, corresponding respectively to no re-experiencing and to very vivid re-experiencing) and then to provide some additional vivid specific details such as thoughts and feelings associated to a specific instant to prove the ability of mentally re-experiencing a specific moment during the navigation.

##### Visuo-spatial recall test

In order to test the memory of the visuo-spatial combination of the “what,” the “where,” and the “when” components, participants were asked, immediately after the free recall, to locate scenes (with associated elements) on a real map supplied by the experimenter for 5 min. Each participant saw a map of their own itinerary.

##### Delayed free recall test

Twenty minutes after the Visuo-spatial recall test, the participants were asked to verbally report again the different elements of the scenes encountered during their navigation (what) and indicate for each of them the different (details, where, when) components (for 10 min).

##### Recognition test

After the delayed free recall test, participants underwent a brief visual recognition test. A series of 10 images were shown on a computer screen to test recognition for the item of information (“what”). For each image, the participants were shown two snapshots of scenes. They had to decide which of the two displayed scenes was or not in the virtual town. Their response had to be based on the scene, and additionally on elements in the scene and on the spatial location of elements in the scene.

#### Scoring

##### Free recall test

Quantitative scoring was done for each of the 16 scenes viewed in the VE to evaluate event features recollection.

– The recall of *items* (“*what*”) was evaluated out of a possible 57 points, namely 1 point per each of the 16 scenes (e.g., supermarket, post office, and a car accident) and 1 point per each of 41 associated salient elements (e.g., the monkey statue, the bus shelter, and the woman with a punk hairstyle). A percentage of correct responses was calculated by dividing the number of recalled items by the total number of possible items (57).
– “*Binding*” recall: For each recalled scene (“what”), we noted if the participants recalled associated components (“*perceptual details*,” “*where*,” and “*when*”). For instance, if they recalled the scene “I saw a car accident,” we recorded whether perceptual details were associated with it (e.g., “one of the cars was yellow;” “there was a women with brown hair who witnessed the accident”), as well as recalls of where and when they saw it (e.g., “I turned left just after seeing the car accident,” “It happened after I passed the supermarket”). A score of bn corresponded to the number of times the recall of a scene was associated with additional *n* relevant information; *n* varied from 1 to 3. For instance, a score of b2 corresponded to the number of times two relevant recalls were associated to the *what* recall (e.g., temporal and spatial recalls). For each bn score, we calculated a binding percentage by dividing the number of correct bindings by the total of scenes (what). We also calculated a total immediate and delayed binding score by adding all the *n* information divided by the number of scenes.
– Thanks to the Remember/Know paradigm, we obtained a score of intensity of subjective sense of remembering (R) in terms of percentage (score on the analogical scale divided by the maximum of five) and a score of justified sense of Remembering (justified R) by taking into account the percentage of R responses justified by the recall of internal details such as thoughts and feelings associated to specific experiences during navigation.

##### Visuo-spatial test

The location of elements on a real map was scored based on the number of correct element locations on the map (out of 57). A percentage was calculated by dividing the number of items correctly located by the maximum number of elements (57).

##### Recognition task

A total percentage was calculated by dividing the number of correct responses by the total number of questions (10).

## Results

### Brief cognitive assessment

First, a series of analyses of variance (ANOVAs) with Age and Condition as between-subject factors, followed by *post hoc* Tukey tests, was performed on the scores of the brief cognitive assessment to ensure that the participants were well matched across the four conditions. For the memory scores, we obtained an effect of age, but no effect of condition, or interaction. The older participants were also well matched across the four conditions concerning their cognitive flexibility and subjective memory complaints (see Table 1).

### Experimental memory VR assessment (VR–EM test)

A series of two-way ANOVAs was conducted with Age and Condition as the between-subject factors for each measure of the VR–EM test (see Table 2). Moreover, to test the effect of delay of retention on free verbal recall scores directly, we carried out mixed ANOVAs with Age and Condition as the between-subject factors, and Delay as within-subject factor on the *what* and the *binding* scores. Finally, we ran a mixed ANOVA with Age and Condition as the between-subject factors, and Delay and Level of binding (b1, b2, and b3) as within-subject factors. The effect sizes were represented with partial eta squared (η^2^). In agreement with Guéguen (2009), we considered effect sizes as small for η^2^ < 0.06, medium for 0.06 ≤ η^2^ < 0.14, and large for η^2^ ≥ 0.14. To determine the direction of the differences, we carried out *post hoc* Tukey tests. We also carried out correlations between VR binding scores and neuropsychological scores, then controlling for age.

**Table 2:** **Mean and SD of the VR–MEM test according to the age-group and the experimental condition and results of the ANOVAs**.

	Score	Experimental conditions								ANOVAs		
		Passive (1)		Itinerary control (2)		Low navigation control (3)		High navigation control (4)		Age effect	Condition effect	Interaction
		YA	OA	YA	OA	YA	OA	YA	OA	F(1,120)	F(3,120)	F(3,120)
	Duration of the navigation (s)	327.75 (52.99)	366.50 (40.22)	350.50 (43.73)	379.68 (51.07)	350.37 (89.91)	314.00 (45.57)	366.50 (85.54)	380.94 (85.24)	1.00η^2^ = 0.00	2.61^t^η^2^ = 0.06	2.12η^2^ = 0.05
Verbal free recall	What (% Immediate)	21.38 (6.84)	18.31 (7.70)	25.88 (9.98)	22.25 (5.54)	25.71 (6.63)	19.18 (6.98)	19.41 (5.38)	16.99 (8.19)	11.52***η^2^ = 0.09	5.60**^a^η^2^ = 0.13	0.37η^2^ = 0.00
	What (% Delayed)	25.76 (7.07)	17.87 (6.67)	29.00 (10.93)	23.35 (6.74)	28.07 (8.44)	19.73 (6.81)	23.79 (7.27)	18.42 (7.52)	24.98***η^2^ = 0.18	3.29*^b^η^2^ = 0.08	0.32η^2^ = 0.00
	Binding (% Immediate)	29.17 (10.20)	19.01 (10.33)	34.11 (14.10)	25.65 (9.21)	34.38 (7.45)	20.96 (7.52)	25.65 (6.02)	18.10 (9.49)	31.02***η^2^ = 0.21	5.65***^c^η^2^ = 0.13	0.45η^2^ = 0.01
	Binding (% Delayed)	30.86 (11.56)	18.62 (6.61)	36.46 (13.13)	23.57 (9.43)	37.37 (12.13)	19.40 (8.29)	27.47 (7.99)	17.19 (9.29)	53.69***η^2^ = 0.32	3.54*^d^η^2^ = 0.08	1.23η^2^ = 0.03
	Remember (%)	58.75 (11.47)	56.25 (16.33)	65.00 (13.66)	66.25 (12.04)	58.75 (13.60)	60.00 (16.68)	61.25 (11.47)	58.13 (10.46)	0.04η^2^ = 0.00	2.06η^2^ = 0.05	0.18η^2^ = 0.00
	Justified R (%)	36.43 (23.54)	21.70 (23.55)	18.23 (21.81)	38.40 (23.95)	34.29 (25.75)	30.09 (26.08)	32.06 (23.35)	20.25 (25.79)	0.71η^2^ = 0.00	0.35η^2^ = 0.00	3.27*^f^η^2^ = 0.08
Visuo-spatial recall	Location (on a real map)	10.85 (4.13)	7.01 (4.45)	13.81 (6.49)	7.89 (5.20)	13.59 (5.05)	7.73 (3.93)	8.88 (4.13)	6.25 (3.74)	28.20***η^2^ = 0.20	4.86^e^η^2^ = 0.11	1.46η^2^ = 0.04
Recognition	Total score (%)	73.75 (10.24)	66.25 (12.04)	70.00 (15.95)	69.37 (9.97)	72.37 (11.17)	66.25 (18.21)	73.75 (16.68)	59.12 (19.73)	6.84**η^2^ = 0.06	0.54η^2^ = 0.01	1.62η^2^ = 0.04

ANOVA age × condition: ***p < 0.001, **p < 0.01, *p < 0.05, ^t^*p* ≤ 0.06.

**Post hoc* Tukey pairwise comparisons on condition effect*.

^a^1 < 2*, 2 > 4**, 3 > 4*.

^b^1 < 2^t^, 2 > 4*.

^c^1 < 2* & 3*, 2 > 4**, 3 > 4*.

^d^1 < 2^t^, 2 > 4*, 3 > 4^t^.

^e^1 < 2* & 3^t^, 2** & 3* > 4; and condition × age effect.

^f^OA: 1 < 2*, 2 > 4*, YA: 1 = 2 = 3 = 4.

### Free verbal recall scores

All the results are presented in Table 2. The effect of Condition in both age-groups concerning the duration of navigation was marginally significant (*p* = 0.05, η^2^ = 0.06) but a *post hoc* pairwise Tukey test indicated no significant differences.

#### Items information

The ANOVA_RM_ Group × Condition × Delay showed a significant main effect of Group [*F* (1,119) = 20.36, *p* < 0.001, η^2^ = 0.15], of Condition [*F* (1,119) = 4.83, *p* < 0.01, η^2^ = 0.11], and of Delay [*F* (1,119) = 24.54, *p* < 0.001, η^2^ = 0.17], as well as a significant Delay × Group [*F* (1,119) = 8.08, *p* < 0.01, η^2^ = 0.07] interaction. The young adults performed better than the older adults and *post hoc t*-tests indicated that the participants in the IC condition achieved a better performance than those in the Passive (*p* < 0.05) and the HNC (*p* < 0.01) conditions (25.12 vs. 21.50 and 19.65%). The participants in the LNC condition performed better than those in the HNC (*p* < 0.05) condition (22.65 vs. 19.65%). The overall performance of the younger group was greater after delayed than immediate recall (Delayed vs. Immediate: 26.66 vs. 23.09%, *p* = 0.001), whereas the performance did not differ between the two recalls in the older group (Delayed vs. Immediate: 19.96 vs. 19.23%, *p* > 0.10). Neither the Delay × Condition nor the Delay × Group × Condition interactions were significant [*F* (1,119) < 1, *p* > 0.10, η^2^ < 0.02 for both interactions]. Thus, the effects of Condition on *what* performance did not vary according to the delay and the age.

#### Binding

The ANOVA_RM_ Group × Condition × Delay showed a significant main effect of Group [*F* (1,119) = 46.51, *p* < 0.001, η^2^ = 0.29], of Condition [*F* (1,119) = 4.99, *p* < 0.01, η^2^ = 0.11], of Delay [*F*(1,119) = 13.50, *p* < 0.001, η^2^ = 0.10], and a significant Delay × Group [*F*(1,119) = 11.39, *p* < 0.01, η^2^ = 0.09] interaction. *Post hoc* tests indicated that participants who had navigated in the IC condition performed better than participants who had navigated in the Passive (*p* < 0.05) and HNC (*p* < 0.01) conditions (29.94 vs. 24.06 and 22.46%), and that participants who had navigated in the LNC condition performed better than those who had navigated in the Passive and the HNC (27.83 vs. 24.06%, *p* = 0.06, and vs. 22.46%, *p* < 0.05). The overall performance of the younger group was greater after delayed than immediate recall (Delayed vs. Immediate: 33.04 vs. 30.82%, *p* < 0.05), whereas the performance did not differ between the two recalls in the older group (Delayed vs. Immediate: 19.69 vs. 20.93%, *p* > 0.10). Neither the Delay × Condition, nor the Delay × Group × Condition interactions were significant [*F* (1,119) < 1, *p* > 0.10, η^2^ = 0.01 or η^2^ = 0.03]. Thus, the effects of Condition on binding performance did not vary according to the delay and the age.

The ANOVA_RM_ Group × Condition × Delay × Level of binding (b1, b2, and b3) showed in addition a main effect of the Level of binding [*F* (2,238) = 48.76, *p* < 0.001, η^2^ = 0.30], and a Level of binding × Group interaction [*F* (2,238) = 16.42, *p* < 0.0001, η^2^ = 0.12]. *Post hoc* tests indicated that for the young group, the percentage of b2 was superior to the percentage of b1 and b3 (b1: 5.59%, b2: 21.09%, b3: 15.69%, *p* < 0.0001), and b3 was superior to b1 (*p* < 0.0001). For the older group, the percentage of b2 was superior to the percentage of b1 and b3 (b1: 9.56%, b2: 15.57%, b3: 6.73%, *p* < 0.0001), and b1 was superior to b3 (*p* < 0.05). Between-group comparisons indicated a performance difference in favor of the older group for b1 (*p* < 0.05), and in favor of the younger group for b2 and b3 (*p* < 0.0001).

**Figure 3:**
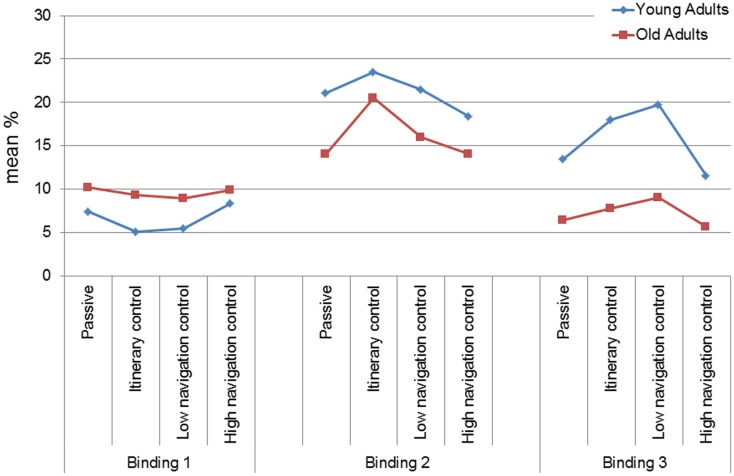
**Main effects of condition (mean and standard deviation) for the mean immediate and delayed binding score in the VR–EM test according to the level of binding and age-group**.

Finally, the Level of binding × Condition [*F* (6,238) = 3.39, *p* < 0.05, η^2^ = 0.07] interaction was significant (see Figure 3). The binding profile differed between participants who navigated in the Passive (b2 > b1, *p* < 0.0001; b2 > b3, *p* < 0.01, b1 = b3) and HNC (b2 > b1, *p* < 0.05; b2 > b3, *p* < 0.01, b1 = b3) conditions and those who navigated in the IC (b2 > b3 & b1, *p* < 0.0001; b3 > b1, *p* < 0.05) and LNC (b2 > b1, *p* < 0.0001, b3 > b1, *p* < 0.05, b2 = b3) conditions. In other words, while there was no effect of Condition for b1, there was an effect of Condition for b2 and b3 (for b2: IC > Passive and LNC, *p* < 0.05, IC > HNC, *p* < 0.05; for b3: IC = LNC > HNC = Passive, *p* < 0.05).

#### Remember responses

The ANOVA (see Table 2) indicated that no simple effect of Age or Condition nor interaction was found for the Remember score, but an interaction was found for the justified Remember score. Older participants in the IC condition gave a higher percentage of justified R responses compared to Passive and HNC conditions (*p* < 0.05), while there was no difference between IC and LNC, and LNC and HNC. There was no difference between the conditions for the younger group.

### Visuo-spatial score

The ANOVA on the score concerning the location of elements (see Table 2) indicated a decrease with aging and a significant effect of the condition regardless of the group. Those who were in the IC and the LNC conditions performed better than participants who were in the Passive (*p* < 0.05 and *p* < 0.06) and HNC condition (*p* < 0.01 and *p* < 0.05). There was no other difference.

### Recognition

A decrease of performance with aging was observed for the total recognition score (YA vs. OA: 72.47 vs. 65.00%). There was no effect of condition or interaction (see Table 2).

### Correlation between VR–EM test and neuropsychological tests

As illustrated in Table 3, the mean VR binding score was significantly correlated with visual memory (except for the passive condition) and working visuo-spatial memory (including short-term binding). In addition, VR binding scores were correlated with executive function (shifting) for low and HNC conditions. When controlling for age, all significant correlations vanished except for the HNC. Indeed, the VR binding scores of participants who navigated in the HNC condition still remained correlated with visual memory, working memory, and executive function. The partial correlation between binding scores and executive function remained significant after application of the Bonferroni correction.

**Table 3:** **Correlations between VR binding mean scores and neuropsychological tests as a function of condition (*n* = 36 per condition)**.

	Age	Verbal memory	Visual memory	Working memory	Executive functions
Passive	−0.47**	0.10	0.29	0.43**	−0.31
Itinerary control	−0.41*	0.33*	0.52** (0.37*)	0.33*	−0.27
Low navigation control	−0.72***	0.31	0.62***	0.67***	−0.43**
High navigation control	−0.46**	0.32	0.53*** (0.37*)	0.55*** (0.42*)	−0.58*** (−0.48**)

*VR binding mean scores correspond to the mean of immediate and delayed binding scores; for neuropsychological measures (see Table 1 and brief cognitive assessment)*.

*Correlations in brackets remain significant after controlling for age*.

***p < 0.001; **p < 0.01; *p < 0.05.

*Correlations in bold remain significant after application of the Bonferroni correction of *p* = 0.002*.

## Discussion

Using a naturalistic environment created with VR, the present study aimed to assess the distinctive role of decision or motor control on feature binding, and to illuminate the relationships between binding, form of encoding, and aging in order to suggest new procedures that could improve feature binding by focusing on the influence of action at encoding. We manipulated the amount of active navigation and decision of the itinerary while younger and older participants navigated in a virtual town trying to memorize all the events they experienced. We then assessed EM (e.g., what–where–when feature binding) via a series of immediate and delayed verbal and visuo-spatial tests. Our main findings showed that both LNC and the choice of the itinerary (IC) enhanced EM performance in young and older participants. By contrast, HNC and passive navigation did not help EM performance in the two age-groups. The role of action, either active navigation or decision, in EM is discussed as well as its influence in aging.

The benefit of active navigation on EM performance depended on the amount of active control (high vs. low control) regardless of the age. Although it is generally agreed that action at encoding enhances memory by enriching memory traces in laboratory settings (Engelkamp, 1998; Madan and Singhal, 2012; Zimmer and Cohen, 2001), and that increasing body-based interaction (i.e., translational and rotational body-based interaction) in participants who navigate through a VE generally improves spatial performances (Ruddle and Lessels, 2009; Ruddle et al., 2011), sensorimotor interaction in VEs, compared with no interaction, has not been always associated with better memory performance, especially for factual information (objects, elements, and scenes encountered, see also the Section “Introduction”). We postulated that when active navigation control is too demanding for participants’ cognitive resources (Gaunet et al., 2001; Chrastil and Warren, 2012) it would not help EM performance. Consistent with this hypothesis, we clearly found that the HNC condition (i.e., ordinary drive-like manipulation of a steering-wheel and pedals) was generally the worst way (similar to the passive condition) to memorize episodic features from complex naturalistic scenes in our virtual setting. This damaging effect was observed whatever the VR measures (free verbal recall of What and Binding information, free recall of visuo-spatial information), except for the recognition of What information where no effect of condition was observed. In fact, HNC, like the passive condition, did not help young or older participants’ high levels of binding (i.e., three pieces of information), contrarily to IC and LNC conditions. Our hypothesis was that HNC (controlling both the pedals and the steering-wheel in a complex VE) differed in the degree of interactive motor engagement but also in the degree of executive/attentional load compared to LNC. This was substantiated by correlational analyses revealing that memory performance during HNC was mainly related to executive function regardless of the age of the participants. Thus, these findings seem to confirm that HNC acted as a divided attention condition that required a higher level of attentional resources, regardless of the age of participants (Craik et al., 1996; Anderson et al., 1998; Naveh-Benjamin et al., 2005), which, in turn, impeded memory function.

By contrast, the effective role of low active motor control resulted in significantly enhanced EM. Importantly here, we confirm a benefit of active navigation control, compared to passive navigation, not only for visuo-spatial recall, as did previous studies (Brooks et al., 1999; Péruch and Wilson, 2004; Wallet et al., 2011; Plancher et al., 2012, 2013), but also for *item memory* (i.e., What scores: scenes/events with perceptible details) and *binding* scores (i.e., scenes/events situated in their specific spatiotemporal context). Interestingly, this positive effect was larger for the binding scores than for the What scores (e.g., no effect was noted for What delayed scores). LNC in particular helped young and older participants’ high levels of binding (i.e., three pieces of information). The findings regarding What scores are in line with one study showing that item memory for objects placed in the rooms of a virtual apartment was enhanced by active (via a joystick) compared to passive navigation (Sauzéon et al., 2011), and contradict some other results showing no effect on item memory (Brooks et al., 1999; Plancher et al., 2013). They also confirmed previous results in aging (Plancher et al., 2012) where active navigation in VEs (as the driver of a car) yielded a better recall of the item memory and spatial information, as well as binding in comparison to passive navigation (as the passenger of the car). Therefore, the present study did not confirm the assumption that active navigation would be helpful for the encoding of spatial information (that is directly targeted by the action), but negative for the encoding of items (because indirectly related to the action proper) (Brooks et al., 1999; Plancher et al., 2013). This divergence may result from differences in the experimental designs (Wallet et al., 2011; Chrastil and Warren, 2012). For instance, instead of a joystick, active navigation used here a higher body-based interaction with the VE, asking participants to control the speed and the stops with pedals, not the turns. In this way, driving the virtual car was unlikely to prove difficult, especially when cornering (where most of the scenes and events were situated). Therefore, the LNC condition did not appear to require a higher level of attentiveness, unlike HNC, and interestingly this condition seemed to add an environmental support at encoding, even for the elderly [see Luo and Craik (2008) for a review], via motor action and driver assistance (Blankertz et al., 2010). Moreover, instructions asked for intentional encoding of elements and events with as much detail as possible, including spatiotemporal situation and perceptive details, not only for objects and spatial layout. Interestingly, the difference in the LNC effect according to the What and binding scores could explain the contradictory results regarding the effect of active navigation on subsequent memory, as binding memory measures are more sensitive to the enactment effect of active navigation. For instance, no effect of active navigation (compared to passive navigation) was found in several studies using spatial memory measures (Wilson, 1999; Gaunet et al., 2001; Sandamas and Foreman, 2003; Foreman et al., 2005; Taillade et al., 2013), or item memory measures (Brooks et al., 1999; Plancher et al., 2013), whereas such an effect was previously found using feature binding measures of associated what–where–when information (Plancher et al., 2012). All things considered, we can assume that after active motor control, when the task does not require too high a level of cognitive control (see Discussion above), memory of the scenes/events encountered in VE are enhanced because they are enriched by a motor trace at encoding that provides specific cues at retrieval (Engelkamp, 1998; Nilsson et al., 2000; Nyberg et al., 2001). As a result, it is suggested that in large-scale naturalistic VEs, possibly like in everyday memory, motor interaction can help integrated information with item-specific and associative information (Eichenbaum, 2000; Atienza et al., 2011).

The most interesting and novel finding of the present study is that decision of the itinerary (IC) without any physical activity was remarkably effective in boosting EM in both young and older participants. Although deciding has been related to executive/frontal functions (MacPherson et al., 2002; Denburg et al., 2007), this was the best condition for enhancing EM. This benefit was observed at immediate and delayed free verbal recall and visuo-spatial recall (IC was generally significantly better than Passive and HNC, and similar to LNC), leading to the best levels of binding. The crucial role of decision on memory encoding in virtual navigation is in line with previous assumptions (Wilson, 1999; Bakdash et al., 2008; Chrastil and Warren, 2012; Plancher et al., 2013). As far as we know, only two previous experimental studies have been published. In the VR study by Bakdash et al. (2008), the effect of decision-making was compared to motor control on subsequent spatial memory of young adults, but there was no passive condition. Performance was better when the VE was learned with decision-only (similar to our IC condition, the participant decided where to go by giving verbal directions to the experimenter) or decision plus motor control, compared to when only motor control was present (the participant only had joystick control, the experimenter instructed participants where to go). Using an experimental design very similar to the present study, Plancher et al. (2013) found that decision-only on the itinerary enhanced subsequent item and spatial memory, compared to passive navigation, while motor control-only benefited spatial memory. The present study therefore provides new evidence for the positive influence of decision-making on what–where–when feature binding.

How can we explain this benefit of decision-making on EM? This condition did not create body-based information of actions like HNC and LNC, only subject-directed activity. The finding here can nicely contribute to the debate on the mechanisms responsible for the enactment effect (using SPT or active navigation). The SPT literature attributes the enactment effect either to the multimodal nature of a motor action (Engelkamp, 1998) or to the involvement of subject-directed activity rather than motor activity *per se* (Kormi-Nouri, 1995). According to some authors (Wilson, 1999; Bakdash et al., 2008; Chrastil and Warren, 2012), the effect of active navigation on subsequent memory might be due to sensorimotor activity and the subject’s directed activity (planning, decision-making, and attention). Neglecting to disentangle these two facets might explain the inconsistent results concerning the active–passive effect during virtual navigation. In the present study, the decisional condition allowed participants to have intentional control over the perceived environment during encoding by making right–left turn decisions where most of the scenes were situated. Thus, it appears that planning or deciding an action directly associated with a scene was particularly efficient in implementing binding processes representing the features of the action and scene, creating the representation of a personal event. As highlighted by Hommel (2004), integrating multimodal codes are important for binding, and applies not only to sensorimotor processing but also to action planning. In the same vein, Voss et al. (2011) suggested that “volitional control” may improve the performance in memory thanks to the interplay between distinct neural systems related to planning, attention, and object processing. They argued that such control improves EM performance because the hippocampus is not only concerned by relational feature binding (Eichenbaum, 2000; Ergorul and Eichenbaum, 2004), but also by planning (Bird and Burgess, 2008; Viard et al., 2011). Moreover, binding processes involved in the IC condition could be more or less automatic (Van Asselen et al., 2006) mainly related to hippocampal processes (performance after IC correlated with visual memory performance) providing effective cues at retrieval rather than related to executive/frontal control processes (there was no correlation with executive function). We postulate that deciding the itinerary and memorizing the scenes benefits from multimodal coding: e.g., specific information based on deciding the itinerary, imagining the action (cornering), and viewing the imagined/decided environment (comparing expectations with actual scenes). Imagining a subject-directed activity or imagining personal future events (Buckner and Carroll, 2007; Schacter et al., 2007; Maguire and Hassabis, 2011; Viard et al., 2012) depends to a very large extent on the same neural network as real personal actions or events. Along the same lines, Bakdash et al. (2008) consider that the positive effect of IC may be related to the use of both egocentric and allocentric representations (i.e., local and global spatial information) of the environment, whereas motor control may be achieved using just an egocentric representation. Egocentric frames of reference specify route knowledge of spatial layout from the perspective of a ground-level observer (e.g., eye and body coordinates) navigating the environment and storing sequences of combinations of scenes. Allocentric frames of reference specify survey knowledge characterized by an external perspective, independently of the viewer’s position, allowing direct access to the global spatial layout. Further studies need to determine the most crucial strategies that determine the enactment effect via IC in virtual navigation (Dahmani and Bohbot, 2014).

Regarding the benefit of enactment in aging, even if we reported a benefit of IC and LNC on memory in both young and older adults, it must be acknowledged that these encoding conditions did not fully compensate for age-related effects on EM (i.e., no interaction between condition and age was generally observed). This pattern has been found by manipulating other factors known to enhance memorization such as self-reference processing (Gutchess et al., 2007; Lalanne et al., 2013) or self-performed tasks (Feyereisen, 2009) during encoding: enhanced performances are usually reported in aging, but older people still perform more poorly than young adults. Regardless of the condition, a decrease in performance with aging was found for event feature bindings and we showed that the age-related decline was larger for the *binding* scores than for the *what* scores, i.e., larger for associative memory than for item memory (Parkin and Walter, 1992; Kessels et al., 2007; Mitchell and Johnson, 2009). More precisely, we found that older adults had greater difficulty in binding several pieces of information with event content compared to young adults (i.e., binding with just one piece of information). The present study confirms this point with a naturalistic paradigm, highlighting that the binding difficulties in the elderly came from encoding. Indeed, free recall performance remained stable after a retention time of 20 min, while the performance of young adults improved, and age decline was persistent in recognition. These age-related effects on feature binding were generally mediated by verbal (for IC) and visual memory (for IC, LNC, and HNC), working memory including short-term binding (for Passive, IC, LNC, and HNC), and executive function (for LNC and HNC). This is in line with previous findings in aging demonstrating that age decline in VR navigation is mediated by composite processes including episodic and working memory (Gyselinck et al., 2013), and executive function (Taillade et al., 2013), confirming age-related effects on both the *associative* and *strategic* components of EM (Moscovitch, 1992; Shing et al., 2008).

Interestingly, in older participants the IC condition was able to boost feature binding and the capacity to mentally re-experience the original navigation, providing personal details of a specific moment. Both capacities are generally altered in aging (Parkin and Walter, 1992; Spencer and Raz, 1995; Piolino et al., 2006; Mitchell and Johnson, 2009). A subject-directed activity in the IC condition thus seems able to reduce age-related EM deficits (compared to other conditions) by enhancing both the subjective (i.e., sense of remembering or autonoetic consciousness, Tulving, 2002) and objective aspects (i.e., contextual information) of specific events memory. Moreover, although participants in each condition experienced similar first-person visual information (i.e., egocentric VR exposure in the encoding phase), which is consider to alter spatial memory in aging (Morganti and Riva, 2014), it may be suggested that the IC condition induced the use of specific reference frames such as allocentric representations (Bakdash et al., 2008), which have no impact on spatial memory in the elderly. Future research is needed to investigate this important issue (Ruggiero et al., 2009). This suggests that the IC condition may result in enhanced EM performance in older adults by supporting multiple memory aspects including allocentric representations (Morganti and Riva, 2014), verbal processes (Brickman and Stern, 2009), hippocampal-related processes (Voss et al., 2011), and future-oriented behaviors (Buckner and Carroll, 2007; Schacter et al., 2007).

Finally, despite its promising results, the present pilot study has a few limitations that further research will be able to overcome. First, the sample size of groups was rather small (16 participants for each Condition × Age). Nevertheless, size effects were generally large for age and medium for condition. Second, we used intentional encoding, while real-life is generally concerned by incidental encoding. In a previous study, we showed that the age-related difference was similar for feature binding with both types of encoding (Plancher et al., 2010), but it would be interesting to further investigate the effect of navigation or IC using incidental encoding. Moreover, since elderly memory performance is inherent in egocentric and/or allocentric strategies on navigational tasks (Morganti and Riva, 2014), our findings might be partly dependent on the use of an egocentric VR exposure. This domain of research could be interestingly extended to the comparison between healthy aging and Alzheimer’s disease since the former is more specially concerned by egocentric encoding (Iachini et al., 2009b; Morganti and Riva, 2014) while the latter is more concerned by allocentric encoding (e.g., map and GPS), or transfer from allocentric to egocentric representations (Morganti et al., 2013; Morganti and Riva, 2014; Serino et al., 2014). We can expect the IC condition to be less effective than LNC in patients with Alzheimer’s disease, unlike in healthy aging. A further important issue would be to substantiate the findings using different reference frames (Committeri et al., 2004; Avraamides and Kelly, 2008; Ruggiero et al., 2009). It will be particularly interesting to test the impact of decision-making or motor control by contrasting allocentric and egocentric strategies on feature binding. Finally, future research should also include a condition in which participants are both active in navigation and decision on the itinerary. We did not plan this condition here because it is the one that was generally addressed in previous VR studies on spatial memory and that gave conflicting results. Our objective in the present study was therefore to distinguish the two components to clarify the pattern of results on effect of action in VE regarding EM performances, especially as regards feature binding. However, while we have shown that both LNC and IC can boost EM, further research should investigate the effect of combining the two as done by Bakdash et al. (2008) on spatial performances. Although these authors did not find any differences between decision-only and decision *plus* motor control conditions, it can be assumed that this combined condition (LNC *plus* IC) could help feature binding more than the two conditions separately, and could in this case reduce the difference between young and old.

In conclusion, the novelty of this study was to highlight the benefit of both IC and LNC in EM performance in young and older adults, emphasizing the advantageous influence on long-term feature binding. While this research needs to be continued to strengthen its conclusions, the initial findings suggest that navigational and decisional activity during real-life events should be useful in aging to boost EM. It could encourage older adults to use their own actions, both via active navigation and decisional control, to boost the encoding of complex events in their daily life. Moreover, it could be useful for EM training programs in aging and patients with authentic EM deficits due to encoding impairment, such as Alzheimer’s disease. Our study offers new insights into the relationship between EM, different action related processes, and aging, and opens up new avenues of research in this area and training programs. Indeed, looking at the conditions under which older adults’ EM can be enhanced, using conditions somewhat similar to real-life settings that allow for increased interaction with the environment is an important issue for future research.

## Conflict of Interest Statement

The authors declare that the research was conducted in the absence of any commercial or financial relationships that could be construed as a potential conflict of interest.

## References

[B1] Albert M. (1994). “Age-related changes in cognitive function,” in Clinical Neurology of Aging, eds Albert M. Knoefel J. (New York, NY: Oxford University Press), 314–326.

[B2] Anderson N. D. Craik F. I. M. Naveh-Benjamin M. (1998). The attentional demands of encoding and retrieval in younger and older adults: 1. Evidence from divided attention costs. Psychol. Aging 13, 405–423. 10.1037/0882-7974.13.3.405 9793117

[B3] Arvind-Pala P. N’Kaoua B. Mazaux J. M. Simion A. Lozes S. Sorita E. (2014). Everyday-like memory and its cognitive correlates in healthy older adults and in young patients with traumatic brain injury: a pilot study based on virtual reality. Disabil. Rehabil. 9, 463–73. 10.3109/17483107.2014.941952 25030298

[B4] Atienza M. Atalaia-Silva K. C. Gonzalez-Escamilla G. Gil-Neciga E. Suarez-Gonzalez A. Cantero J. L. (2011). Associative memory deficits in mild cognitive impairment: the role of hippocampal formation. Neuroimage 57, 1331–1342. 10.1016/j.neuroimage.2011.05.047 21640840

[B5] Avraamides M. Kelly J. (2008). Multiple systems of spatial memory and action. Cogn. Process. 9, 93–106 10.1007/s10339-007-0188-5 17899235

[B6] Bakdash J. Z. Linkenauger S. A. Profitt D. (2008). Comparing decision-making and control for learning a virtual environment: backseat drivers learn where they are going. Proc. Hum. Fact. Ergon. Soc. Annu. Meet. 52, 2117–2121 10.1177/154193120805202707

[B7] Barra J. Laou L. Poline J. B. Lebihan D. Berthoz A. (2012). Does an oblique/slanted perspective during virtual navigation engage both egocentric and allocentric brain strategies? PLoS ONE 7:e49537. 10.1371/journal.pone.0049537 23209583 PMC3509118

[B8] Berthoz A. (2003). La Décision, ed. Jacob O. (Paris).

[B9] Bird C. M. Burgess N. (2008). The hippocampus and memory: insights from spatial processing. Nat. Rev. Neurosci. 9, 182–194 10.1038/nrn2335 18270514

[B10] Blankertz B. Tangermann M. Vidaurre C. Fazli S. Sannelli C. Haufe S. (2010). The Berlin brain–computer interface: non-medical uses of BCI technology. Front. Neurosci. (2010) 4:198 10.3389/fnins.2010.00198 21165175 PMC3002462

[B11] Bohbot V. D. McKenzie S. Konishi K. Fouquet C. Kurdi V. Schachar R. (2012). Virtual navigation strategies from childhood to senescence: evidence for changes across the life span. Front. Aging Neurosci. 4:28. 10.3389/fnagi.2012.00028 23162463 PMC3498879

[B12] Bohil C. J. Alicea B. Biocca F. A. (2011). Virtual reality in neuroscience research and therapy. Nat. Rev. Neurosci. 12, 752–762. 10.1038/nrn3122 22048061

[B13] Brickman A. M. Stern Y. (2009). “Aging and memory in humans,” in Encyclopedia of Neuroscience, Vol. 1 ed. Squire L. R. (Oxford: Academic Press), 175–180.

[B14] Brooks B. M. Attree E. A. Rose F. D. Clifford B. R. Leadbetter A. G. (1999). The specificity of memory enhancement during interaction with a virtual environment. Memory 7, 65–78. 10.1080/741943713 10645373

[B15] Buckner R. L. Carroll D. C. (2007). Self-projection and the brain. Trends Cogn. Sci. 11, 49–57 10.1016/j.tics.2006.11.004 17188554

[B16] Burgess N. Maguire E. A. O’Keefe J. (2002). The human hippocampus and spatial and episodic memory. Neuron 35, 625–641. 10.1016/S0896-6273(02)00830-9 12194864

[B17] Burgess N. Maguire E. A. Spiers H. J. O’Keefe J. (2001). A temporoparietal and prefrontal network for retrieving the spatial context of lifelike events. Neuroimage 14, 439–453 10.1006/nimg.2001.0806 11467917

[B18] Carassa A. Geminiani G. Morganti F. Varotto D. (2002). Active and passive spatial learning in a complex virtual environment: the effect of efficient exploration. Cogn. Process. 3-4, 65–81.

[B19] Chalfonte B. L. Johnson M. K. (1996). Feature memory and binding in young and older adults. Mem. Cognit. 24, 403–416. 10.3758/BF03200930 8757490

[B20] Chrastil E. R. Warren W. H. (2012). Active and passive contributions to spatial learning. Psychon. Bull. Rev. 19, 1–23 10.3758/s13423-011-0182-x 22083627

[B21] Committeri G. Galati G. Paradis A. L. Pizzamiglio L. Berthoz A. Le Bihan D. (2004). Reference frames for spatial cognition: different brain areas are involved in viewer-, object- and landmark-centered judgments about object location. J. Cogn. Neurosci. 16, 1517–1535. 10.1162/0898929042568550 15601516

[B22] Craik F. I. M. (1986). “A functional account of age differences in memory,” in Human Memory and Cognitive Abilities, Mechanisms and Performance, eds Klix F. Hagendorf H. (New York, NY: Elsevier), 409–422.

[B23] Craik F. I. M. Govoni R. Naveh-Benjamin M. Anderson N. D. (1996). The effects of divided attention on encoding and retrieval processes in human memory. J. Exp. Psychol. 125, 159–180. 10.1037/0096-3445.125.2.159 8683192

[B24] Dahmani L. Bohbot V. D. (2014). Dissociable contributions of the prefrontal cortex to hippocampus- and caudate nucleus-dependent virtual navigation strategies. Neurobiol. Learn. Mem. 10.1016/j.nlm.2014.07.002 25038426

[B25] Deltour J. J. (1993). [Mill Hill Vocabulary Scale of J.C. Raven. French Adaptation and Comparative Norms of the Mill Hill and of Standard Progressive Matrices (PM38)]. Manuel Editions l’Application Des Techniques Modernes. Braine-le-Château, Belgium: Editions L’Application des Techniques Modernes SPRL.

[B26] Denburg N. L. Cole C. A. Hernandez M. Yamada T. H. Tranel D. Bechara A. (2007). The orbitofrontal cortex, real-world decision making, and normal aging. Ann. N. Y. Acad. Sci. 1121, 480–498. 10.1196/annals.1401.031 17872394 PMC2246008

[B27] Earles J. L. Kersten A. W. (2002). Directed forgetting of actions by younger and older adults. Psychon. Bull. Rev. 9, 383–388. 10.3758/BF03196297 12120804

[B28] Earles J. L. Kersten A. W. Mas B. B. Miccio D. M. (2004). Aging and memory for self-performed tasks: effects of task difficulty and time pressure. J. Gerontol.B Psychol. Sci. Soc. Sci. 59B, 285–293. 10.1093/geronb/59.6.P285 15576856

[B29] Eichenbaum H. (2000). A cortical-hippocampal system for declarative memory. Nat. Rev. Neurosci. 1, 41–50. 10.1038/35036213 11252767

[B30] Engelkamp J. (1998). Memory for Actions. Hove: Psychology Press.

[B31] Engelkamp J. Zimmer H. D. Mohr G. Sellen O. (1994). Memory of self-performed tasks: self-performing during recognition. Mem. Cognit. 22, 34–39. 10.3758/BF03202759 8035683

[B32] Ergorul C. Eichenbaum H. (2004). The hippocampus and memory for “what,” “where,” and “when”. Learn. Mem. 11, 397–405. 10.1101/lm.73304 15254219 PMC498318

[B33] Fabiani M. Friedman D. (1997). Dissociations between memory for temporal order and recognition memory in aging. Neuropsychologia 35, 129–141. 10.1016/S0028-3932(96)00073-5 9025117

[B34] Feyereisen P. (2009). Enactment effects and integration processes in younger and older adults’ memory for actions. Memory 17, 374–385. 10.1080/09658210902731851 19221926

[B35] Folstein M. F. Folstein S. E. Mc Hugh P. R. (1975). Mini-mental state: a practical method for grading the cognitive state of patients for the clinician. J. Psychiatr. Res. 12, 189–198 10.1016/0022-3956(75)90026-6 1202204

[B36] Foreman N. Stanton-Fraser D. Wilson P. N. Duffy H. Parnell R. (2005). Transfer of spatial knowledge to a two-level shopping mall in older people, following virtual exploration. Environ. Behav. 37, 275–292 10.1177/0013916504269649

[B37] Fuchs P. Moreau G. Berthoz A. Vercher J. L. (2006). Le traité de la réalité virtuelle – Volume 1: L’homme et l’environnement virtuel. Paris: Presses De l’Ecole Des Mines De Paris.

[B38] Galati G. Pelle G. Berthoz A. Committeri G. (2010). Multiple reference frames used by the human brain for spatial perception and memory. Exp. Brain Res. 206, 109–120. 10.1007/s00221-010-2168-8 20186405

[B39] Gardiner J. M. (2001). Episodic memory and autonoetic consciousness: a first-person approach. Philos. Trans. R. Soc. 356, 1351 10.1098/rstb.2001.0955 PMC1088519 11571027

[B40] Gaunet F. Vidal V. Kemeny A. Berthoz A. (2001). Active, passive and snapshot exploration in a virtual environment: influence on scene memory, reorientation and path memory. Brain Res. Cogn. Brain Res. 11, 409–420. 10.1016/S0926-6410(01)00013-1 11339990

[B41] Glisky E. L. (2001). “Source memory, aging, and the frontal lobes,” in Perspectives on Human Memory and Cognitive Aging: Essays in Honor of Fergus Craik, eds Naveh-Benjamin M. Moscovitch M. Roediger H. L. III (New York, NY: Taylor & Francis), 265–276.

[B42] Glisky E. L. Kong L. L. (2008). Do young and older adults rely on different processes in source memory tasks? A neuropsychological study. J. Exp. Psychol. Learn. Mem. Cogn. 34, 809–822. 10.1037/0278-7393.34.4.809 18605870 PMC2504728

[B43] Gras D. Gyselinck V. Perrussel M. Orriols E. Piolino P. (2013). The role of working memory components and visuospatial abilities in route learning within a virtual environment. J. Cogn. Psychol. 25, 38–50 10.1080/20445911.2012.739154

[B44] Guéguen N. (2009). L’importance d’un effet: méthodologie simple de détermination et d’évaluation de l’ effect size. Eur. J. Sci. Res. 38, 20–25. 10.1684/san.2011.0240 21873146

[B45] Gutchess A. H. Kensinger E. A. Schacter D. L. (2007). Aging, self-referencing, and medial prefrontal cortex. Soc. Neurosci. 2, 117–133. 10.1080/17470910701399029 18633811

[B46] Gyselinck V. Meneghetti C. Bormetti M. Orriols E. Piolino P. De Beni R. (2013). Considering spatial ability in virtual route learning in early aging. Cogn. Process. 14, 309–316. 10.1007/s10339-013-0557-1 23536003

[B47] Head D. Isom M. (2010). Age effects on wayfinding and route learning skills. Behav. Brain Res. 209, 49–58. 10.1016/j.bbr.2010.01.012 20085784

[B48] Hommel B. (2004). Event files: feature binding in and across perception and action. Trends Cogn. Sci. 8, 494–500. 10.1016/j.tics.2004.08.007 15491903

[B49] Iachini T. Ruggiero G. Ruotolo F. (2009b). The effect of age on egocentric and allocentric spatial frames of reference. Cogn. Process. 10, 222–224 10.1007/s10339-009-0276-9 19693572

[B50] Iaria G. Palermo L. Committeri G. Barton J. S. (2009). Age differences in the formation and use of cognitive maps. Behav. Brain Res. 196, 187–191 10.1016/j.bbr.2008.08.040 18817815

[B51] Iglói K. Doeller C. F. Berthoz A. Rondi-Reig L. Burgess N. (2010). Lateralized human hippocampal activity predicts navigation based on sequence or place memory. Proc. Natl. Acad. Sci. U. S. A. 107, 14466–14471. 10.1073/pnas.1004243107 20660746 PMC2922562

[B52] Johnson M. K. Hashtroudi S. Lindsay D. S. (1993). Source monitoring. Psychol. Bull. 114, 3–28. 10.1037/0033-2909.114.1.3 8346328

[B53] Kessels R. P. C. Hobbel D. Postma A. (2007). Aging, context memory and binding: a comparison of “what, where and when” in young and older adults. Int. J. Neurosci. 117, 795–810. 10.1080/00207450600910218 17454244

[B54] Klencklen G. Després O. Dufour A. (2012). What do we know about aging and spatial cognition? Reviews and perspectives. Ageing Res. Rev. 11, 123–135. 10.1016/j.arr.2011.10.001 22085884

[B55] Kormi-Nouri R. (1995). The nature of memory for action events: an episodic integration view. Eur. J. Cogn. Psychol. 7, 337–363 10.1080/09541449508403103

[B56] Lalanne J. Rozenberg J. Grolleau P. Piolino P. (2013). The self-reference effect on episodic memory recollection in young and older adults and Alzheimer’s disease. Curr. Alzheimer Res. 10, 1107–1117. 10.2174/15672050113106660175 24156261

[B57] Lambrey S. Amorim M. A. Samson S. Noulhiane M. Hasboun D. Dupont S. (2008). Distinct visual perspective-taking strategies involve the left and right medial temporal lobe structures differently. Brain 31, 523–534. 10.1093/brain/awm317 18178570

[B58] Levine B. Svoboda E. Hay J. F. Winocur G. Moscovitch M. (2002). Aging and autobiographical memory: dissociating episodic from semantic retrieval. Psychol. Aging 17, 677–689. 10.1037/0882-7974.17.4.677 12507363

[B59] Lezak M. D. Howieson D. B. Loring D. W. (2004). Neuropsychological Assessment, 4th Edn Oxford: Oxford University Press.

[B60] Lövden M. Schellenbach M. Grossman-Hutter B. Krüger A. Lindenberger U. (2005). Environmental topography and postural control demands shape aging associated decrements in spatial navigation performance. Psychol. Aging 20, 683–694. 10.1037/0882-7974.20.4.683 16420142

[B61] Luo L. Craik F. I. (2008). Aging and memory: a cognitive approach. Can. J. Psychiatry 53, 346–353. 18616854 10.1177/070674370805300603

[B62] MacPherson S. E. Phillips L. H. Della Sala S. (2002). Age, executive function, and social decision making: a dorsolateral prefrontal theory of cognitive aging. Psychol. Aging 17, 598–609. 10.1037/0882-7974.17.4.598 12507357

[B63] Madan C. R. Singhal A. (2012). Using actions to enhance memory: effects of enactment, gestures, and exercise on human memory. Front. Psychol. 3:507 10.3389/fpsyg.2012.00507 23293612 PMC3536268

[B64] Maguire E. A. Frackowiak R. S. J. Frith C. D. (1997). Recalling route around London: activation of the right hippocampus in taxi drivers. J. Neurosci. 17, 7103–7110. 9278544 10.1523/JNEUROSCI.17-18-07103.1997 PMC6573257

[B65] Maguire E. A. Hassabis D. (2011). Role of the hippocampus in imagination and future thinking. Proc. Natl. Acad. Sci. U. S. A. 108, E39 10.1073/pnas.1018876108 21372268 PMC3060246

[B66] Martinelli P. Anssens A. Sperduti M. Piolino P. (2013). The influence of normal aging and Alzheimer’s disease in autobiographical memory highly related to the self. Neuropsychology 27, 69–78. 10.1037/a0030453 23148495

[B67] McNair D. Kahn R. (1983). “Self-assessment of cognitive deficits,” in Assessment in Geriatric Psychopharmacology, eds Crook T. Ferris S. Bartus R. (New Canaan, CT: Powley), 137–143.

[B68] Mitchell K. J. Johnson M. K. (2009). Source monitoring 15 years later: what have we learned from fMRI about the neural mechanisms of source memory? Psychol. Bull. 135, 638–677. 10.1037/a0015849 19586165 PMC2859897

[B69] Mitchell K. J. Johnson M. K. Raye C. L. Mather M. D’Esposito M. (2000). Aging and reflective processes of working memory: binding and test load deficits. Psychol. Aging 15, 527–541. 10.1037/0882-7974.15.3.527 11014715

[B70] Moffat S. D. (2009). Aging and spatial navigation: what do we know and where do we go? Neuropsychol. Rev. 19, 478–489. 10.1007/s11065-009-9120-3 19936933

[B71] Morganti F. Riva G. (2014). Virtual reality as allocentric/egocentric technology for the assessment of cognitive decline in the elderly. Stud. Health Technol. Inform. 196, 278–284. 24732522

[B72] Morganti F. Stefanini S. Riva G. (2013). From allo- to egocentric spatial ability in early Alzheimer’s disease: a study with virtual reality spatial tasks. Cogn. Neurosci. 4, 171–180. 10.1080/17588928.2013.854762 24251605

[B73] Moscovitch M. (1992). Memory and working-with-memory: a component process model based on modules and central systems. J. Cogn. Neurosci. 4, 257–267. 10.1162/jocn.1992.4.3.257 23964882

[B74] Mueller C. Luehrs M. Baecke S. Adolf D. Luetzkendorf R. Luchtmann M. (2012). Building virtual reality fMRI paradigms: a framework for presenting immersive virtual environments. J. Neurosci. Methods 209, 290–298. 10.1016/j.jneumeth.2012.06.025 22759716

[B75] Naveh-Benjamin M. (2000). Adult age differences in memory performance: tests of an associative deficit hypothesis. J. Exp. Psychol. Learn. Mem. Cogn. 26, 1170–1187 10.1037/0278-7393.26.5.1170 11009251

[B76] Naveh-Benjamin M. Craik F. I. Guez J. Kreuger S. (2005). Divided attention in younger and older adults: effects of strategy and relatedness on memory performance and secondary task costs. J. Exp. Psychol. Learn. Mem. Cogn. 31, 520–537. 15910135 10.1037/0278-7393.31.3.520

[B77] Naveh-Benjamin M. Craik F. I. M. Ben-Shaul L. (2002). Age-related differences in cued recall: effects of support at encoding and retrieval. Aging Neuropsychol. Cogn. 9, 276–287 10.1076/anec.9.4.276.8773

[B78] Naveh-Benjamin M. Guez J. Shulman S. (2004). Older adult’s associative deficit in episodic memory: assessing the role of decline in attentional resources. Psychon. Bull. Rev. 11, 1067–1073. 10.3758/BF03196738 15875977

[B79] Nilsson L. G. Nyberg L. Klingberg T. Aberg C. Persson J. Roland P. E. (2000). Activity in motor areas while remembering action events. Neuroreport 11, 2199–2201. 10.1097/00001756-200007140-00027 10923670

[B80] Nyberg L. Petersson K.-M. Nilsson L.-G. Sandblom J. Åberg C. Ingvar M. (2001). Reactivation of motor brain areas during explicit memory for actions. Neuroimage 14, 521–528. 10.1006/nimg.2001.0801 11467924

[B81] Parkin A. J. Walter B. M. (1992). Recollective experience, normal aging, and frontal dysfunction. Psychol. Aging 7, 290–298. 10.1037/0882-7974.7.2.290 1610518

[B82] Parsons T. D. Rizzo A. A. (2008). Initial validation of a virtual environment for assessment of memory functioning: virtual reality cognitive performance assessment test. Cyberpsychol. Behav. 11, 17–25. 10.1089/cpb.2007.9934 18275308

[B83] Péruch P. Wilson P. N. (2004). Active versus passive learning and testing in a complex outside built environment. Cogn. Process. 5, 218–227 10.1007/s10339-004-0027-x

[B84] Picard L. Cousin S. Guillery-Girard B. Eustache F. Piolino P. (2012). How do the different components of episodic memory develop? Role of executive functions and feature-binding abilities. Child Dev. 83, 1037–1050. 10.1111/j.1467-8624.2012.01736.x 22364311

[B85] Piolino P. Coste C. Martinelli P. Macé A. Quinette P. Guillery-Girard B. (2010). Reduced specificity of autobiographical memory and aging: do the executive and feature binding functions of working memory have a role? Neuropsychologia 48, 429–440. 10.1016/j.neuropsychologia.2009.09.035 19804792

[B86] Piolino P. Desgranges B. Benali K. Eustache F. (2002). Episodic and semantic remote autobiographical memory in ageing. Memory 10, 239–257. 10.1080/09658210143000353 12097209

[B87] Piolino P. Desgranges B. Clarys D. Guillery-Girard B. Isingrini M. Eustache F. (2006). Autobiographical memory and sense of remembering in aging. Psychol. Aging 21, 510–525 10.1037/0882-7974.21.3.510 16953713

[B88] Plancher G. Barra J. Orriols E. Piolino P. (2013). The influence of action on episodic memory: a virtual reality study. Q. J. Exp. Psychol. 66, 895–909. 10.1080/17470218.2012.722657 23025821

[B89] Plancher G. Gyselinck V. Nicolas S. Piolino P. (2010). Age effect on components of episodic memory and feature binding: a virtual reality study. Neuropsychology 24, 379–390. 10.1037/a0018680 20438215

[B90] Plancher G. Nicolas S. Piolino P. (2008). Apport de la réalité virtuelle en neuropsychologie de la mémoire: étude dans le vieillissement. Psychol. NeuroPsychiatr. Vieil. 6, 7–22. 10.1684/pnv.2008.0119 18364292

[B91] Plancher G. Tirard A. Gyselinck V. Nicolas S. Piolino P. (2012). Using virtual reality to characterize episodic memory profiles in amnestic mild cognitive impairment and Alzheimer’s disease: influence of active/passive encoding. Neuropsychologia 50, 592–602. 10.1016/j.neuropsychologia.2011.12.013 22261400

[B92] Rauchs G. Orban P. Balteau E. Schimdt C. Degueldre C. Luxen A. (2008). Partially segregated neural networks for spatial and contextual memory in virtual navigation. Hippocampus 18, 503–518. 10.1002/hipo.20411 18240326

[B93] Ruddle R. A. Lessels S. (2009). The benefits of using a walking interface to navigate virtual environments. ACM Trans. Comput. Hum. Interact. 16, 5 10.1145/1502800.1502805

[B94] Ruddle R. A. Volkova E. Mohler B. Bulthoff H. H. (2011). The effect of landmark and body-based sensory information on route knowledge. Mem. Cognit. 39, 686–699. 10.3758/s13421-010-0054-z 21264583

[B95] Ruggiero G. Iachini T. Ruotolo F. (2009). “Spatial memory: the role of egocentric and allocentric frames of reference,” in Spatial Memory: Visuospatial Processes, ed. Thomas J. B. (Nova Science Publishers, Inc), 1–25.

[B96] Sandamas G. Foreman N. (2003). Active and passive spatial learning from a desktop virtual environment in male and female participants: a comparison with guessing controls. J. Health Soc. Environ. Issues 4, 15–22 10.1177/2158244014525424

[B97] Sauzéon H. Arvind Pala P. Larrue F. Wallet G. Déjos M. Zheng X. (2011). The use of virtual reality for episodic memory assessment: effects of active navigation. Exp. Psychol. 59, 99–108. 10.1027/1618-3169/a000131 22044787

[B98] Schacter D. L. Addis D. R. Buckner R. L. (2007). Remembering the past to imagine the future: the prospective brain. Nat. Rev. Neurosci. 8, 657–661. 10.1038/nrn2213 17700624

[B99] Schultheis M. T. Himelstein J. Rizzo A. R. (2002). Virtual reality and neuropsychology: upgrading the current tools. J. Head Trauma Rehabil. 17, 379–394. 10.1097/00001199-200210000-00002 12802250 PMC13667917

[B100] Serino S. Cipresso P. Morganti F. Riva G. (2014). The role of egocentric and allocentric abilities in Alzheimer’s disease: a systematic review. Ageing Res. Rev. 16, 32–44. 10.1016/j.arr.2014.04.004 24943907

[B101] Shing Y. L. Werkle-Bergner M. Li S.-C. Lindenberger U. (2008). Associative and strategic components of episodic memory: a lifespan dissociation. J. Exp. Psychol. Gen. 137, 495–513. 10.1037/0096-3445.137.3.495 18729712

[B102] Simons J. S. Spiers H. J. (2003). Prefrontal and medial temporal lobe interactions in long-term memory. Nat. Rev. Neurosci. 4, 637–648 10.1038/nrn1178 12894239

[B103] Spencer W. D. Raz N. (1995). Differential effects of aging on memory for content and context: a meta-analysis. Psychol. Aging 10, 527–539. 10.1037/0882-7974.10.4.527 8749580

[B104] Taillade M. Sauzéon H. Arvind Pala P. Déjos M. Larrue F. Gross C. (2013). Age-related wayfinding differences in real large-scale environments: detrimental motor control effects during spatial learning are mediated by executive decline? PLoS ONE 8:e67193. 10.1371/journal.pone.0067193 23843992 PMC3699574

[B105] Tulving E. (1985). Memory and consciousness. Can. Psychol. 26, 1–12 10.1037/h0080017

[B106] Tulving E. (2002). Episodic memory: from mind to brain. Annu. Rev. Psychol. 53, 1–25 10.1146/annurev.psych.53.100901.135114 11752477

[B107] Van Asselen M. Van der Lubbe R. H. Postma A. (2006). Are space and time automatically integrated in episodic memory? Memory 14, 232–240. 10.1080/09658210500172839 16484112

[B108] Viard A. Desgranges B. Eustache F. Piolino P. (2012). Factors affecting medial temporal lobe engagement for past and future episodic events: an ALE meta-analysis of neuroimaging studies. Brain Cogn. 80, 111–125. 10.1016/j.bandc.2012.05.004 22705648

[B109] Viard A. Doeller C. F. Hartley T. Bird C. M. Burgess N. (2011). Anterior hippocampus and goal-directed spatial decision making. J. Neurosci. 31, 4613–4621. 10.1523/JNEUROSCI.4640-10.2011 21430161 PMC6622909

[B110] Von Zerssen D. Strain F. Schwarz D. (1974). “Evaluation of depressive states, especially in longitudinal studies,” in Psychological Measurements in Psychopharmacology, ed. Pichot B. (Basel: Karger), 189–202. 10.1159/000395076 4413308

[B111] Voss J. L. Gonsalves B. D. Federmeier K. D. Tranel D. Cohen N. J. (2011). Hippocampal brain-network coordination during volitional exploratory behaviour enhances learning. Nat. Neurosci. 14, 115–122. 10.1038/nn.2693 21102449 PMC3057495

[B112] Wallet G. Sauzéon H. Pala P. A. Larrue F. Zheng X. N’Kaoua B. (2011). Virtual/real transfer of spatial knowledge: benefit from visual fidelity provided in a virtual environment and impact of active navigation. Cyberpsychol. Behav. Soc. Netw. 14, 417–423. 10.1089/cyber.2009.0187 21288136

[B113] Wechsler D. (2000). WAIS-III: Echelle d’Intelligence De Wechsler Pour Adultes, 3ème édition Edn Paris: Les Editions Du Centre De Psychologie Appliquée.

[B114] Widmann C. N. Beinhoff U. Riepe M. W. (2012). Everyday memory deficits in very mild Alzheimer’s disease. Neurobiol. Aging 33, 297–303. 10.1016/j.neurobiolaging.2010.03.012 20392540

[B115] Wilson P. N. (1999). Active exploration of a virtual environment does not promote orientation or memory for objects. Environ. Behav. 31, 752–763 10.1177/00139169921972335

[B116] Wilson P. N. Péruch P. (2002). The influence of interactivity and attention on spatial learning in a desktop virtual environment. Curr. Psychol. Cogn. 21, 601–633.

[B117] Wolbers T. Hegarty M. (2010). What determines our navigational abilities? Trends Cogn. Sci. 14, 138–146. 10.1016/j.tics.2010.01.001 20138795

[B118] Zawadzki J. A. Girard T. A. Foussias G. Rodrigues A. Siddiqui I. Lerch J. P. (2013). Simulating real world functioning in schizophrenia using a naturalistic city environment and single-trial, goal-directed navigation. Front. Behav. Neurosci. 7:180. 10.3389/fnbeh.2013.00180 24324418 PMC3840323

[B119] Zimmer H. D. Cohen R. L. (2001). “Remembering actions: a specific type of memory?,” in Memory for Action: A Distinct form of Episodic Memory?, eds Zimmer H. D. Cohen R. L. Guynn M. Engelkamp J. Kormi-Nouri R. Foley M. N. (New York, NY: Oxford University Press), 3–24.

[B120] Zimmer H. D. Mecklinger A. Lindenberger U. Zimmer H. D. Mecklinger A. Lindenberger U. (2006). Handbook of Binding and Memory: Perspectives from Cognitive Neuroscience. Oxford, NY: Oxford University Press.

